# Late Paleocene paleoclimate recorded in fluid inclusion of halite in Subei basin, East China

**DOI:** 10.1038/s41598-024-66571-3

**Published:** 2024-07-04

**Authors:** Ting Ding, Bing Pan, Hua Zhang, Chenglin Liu, Zhen Yang, M. Santosh

**Affiliations:** 1 School of Earth Science, East China University of Technology, Nanchang, 330013 Jiangxi China; 2 Urumqi Comprehensive Survey Center on Natural Resources, China Geological Survey, Urumqi, 830057 China; 3 Ministry of Natural Resources, Key Laboratory of Metallogeny and Mineral Assessment, Institute of Mineral Resources, Chinese Academy of Geological Sciences, Beijing, 100037 China; 4 China University of Geosciences, Wuhan, 430074 Hubei China; 5 China University of Geosciences (Beijing), Beijing, 10086 China; 6 The University of Adelaide, Adelaide, 5000 Australia

**Keywords:** Halite, Fluid inclusions, Homogenization temperature, Paleoenvironment, Late Paleocene, Subei basin, Palaeoclimate, Geochemistry, Geology, Sedimentology

## Abstract

Salt deposits are indicative of relatively extreme climate events. However, due to insufficient independent temperature proxies, paleotemperature records obtained from salt deposition are still lack. The Paleocene evaporite sequence deposited in the Hongze Depression of Subei Basin of eastern China provides an important terrestrial sediment record during this period. In this study we present total of 488 homogenization temperature (Th) data of halite fluid inclusions from drilling core with different stratigraphic depth after detailed petrological observation. The obtained T_h_ ranged from 17.7 °C to 52.3 °C, with the mean T_h_ value of 34.1 °C that in good agreement with the previous studies of climatic proxies. Our study shows that primary fluid inclusions of halite can serve as a robust tool to construct the ancient earth surface temperature.

## Introduction

The Late Paleocene-Early Eocene is a warm climate period (65–50 Ma). The Paleocene climate was relatively warm, with global deep-sea temperatures about 8 °C higher than today^1^, oxygen isotopes was lower^2^, and atmospheric carbon dioxide concentration similar to today's atmospheric carbon dioxide concentration (390 ppm)^2^. However, with the passage of time, the global deep sea temperature continued to increase and δ^18^O further decreased. Around 55 Ma, the atmospheric CO_2_ concentration began to rise sharply. The ancient air temperature rose sharply in a short period of time. The deep sea temperature exceeded 10 °C. Carbon isotopes and Oxygen isotopes are negatively excursed, but this process lasts for a short time, less than 0.01 Ma^2,3^, and is the Paleocene-Eocene Thermal Maximum (PETM) event^2,4–8^. After the PETM event, the paleotemperature continued to increase and entered the climate optimal period of the Early Eocene. During this period, the δ^18^O and δ^13^C values were negatively excursed, the temperature reached the highest point since the Cenozoic^2,9–11^, and the deep-sea temperature was about 12 °C higher than today^1,12^. The sea level has also reached the highest height since the Cenozoic Era, 50–100 m or even higher than today^6,13^.

Evaporites on the earth surface are a valuable indicator of paleoclimate, chemical composition of ancient water bodies, and biological conditions^14,15^ due to their sensitivity to environment changes. The fluid inclusions formed during the crystallization process of halite recorded the paleoenvironmental information at that time^16^. The homogeneous temperature (Th) of the primary fluid inclusion of halite can reflect the ancient water/air temperature when the halite crystallized^17,18^, and has been widely used to paleo-environmental changes within various salt basin^17,19–21^.

Meng (2011)^20^ and others et al.^22–24^ studied fluid inclusions of halite from modern salt lakes, as well as in halite grown in laboratory conditions, and concluded that primary fluid inclusions in halite exist in funnel crystals formed at the air–water interface and herringbone crystals formed at the bottom of the water. Both, provided the basin is shallow, can be used to reflect the temperature of the ancient environment, and only the maximum uniform temperature is closest to the temperature when the brine crystallized^25^. Zhao^21^ and others^25^ conducted comparative experiments on the homogeneous temperature of a single liquid inclusion in halite rock under different freezing conditions and different high temperature conditions and showed that: without experiencing high temperature (110 °C) and low temperature (− 20 °C) freezing Under interference conditions, the homogenization temperature of a single liquid inclusion in halite obtained at a slow heating rate (usually less than 1 °C/min) can represent the ancient water temperature when the halite was formed.

In this study of the salt-bearing system of the fourth member of the Funing Formation in the Hongze Sag of the Subei Basin, the author found that halite rocks contain a large number of primary fluid inclusions, which provides important materials for studying ancient water temperatures. This article carries out homogeneous temperature analysis on these primary halite inclusions to reveal the paleowater temperature characteristics of the Hongze Sag in the Subei Basin and explore its paleoclimate significance, which is an important reference for studying climate changes in the early Paleogene.

## Study area

### Geological setting

**Figure 1:**
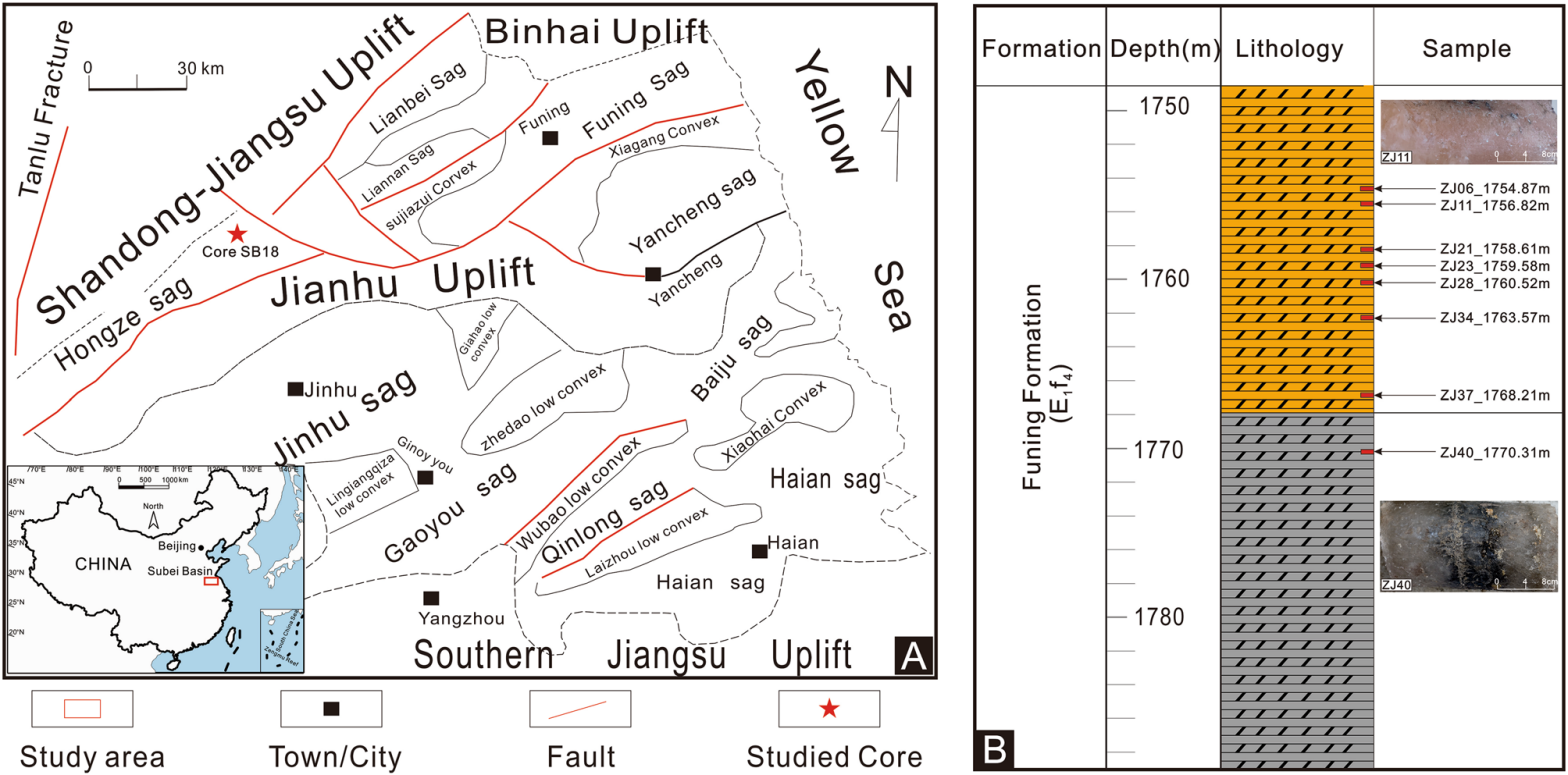
(**A**) Simplified geology of the study area and (**B**) lithology and sample sites of the lower member of Funing Formation in core SB18.

The Subei Basin, located in eastern China, constitutes the onshore section of the North Subei-Yellow Sea Basin. Positioned northeast of the Yangtze Platform, it shares borders with the Binhai Uplift and the Sulu Orogenic Belt to the north, and the South Jiangsu Uplift and the Zhangbaling Uplift to the south. To the west, it is demarcated by the Tanlu Fault Zone. Extending to the Yellow Sea in the east, it spans an area of approximately 4.2 × 10^4^ km^2^. The region boasts numerous structural units, primarily characterized by a succession of asymmetric fault depressions with a "south fault and north superposition^26^ configuration. It follows a fundamental structural layout of secondary basins, forming an east–west axis known as ‘two depressions and one uplift’. From north to south, these are the Yanfu Depression, Jianhu Uplift, and Dongtai Depression (Fig. 1A). Enclosed by the Jianhu uplift at the basin's midpoint, the area northward toward the Binhai uplift is termed the Yanfu Depression, while the region southward toward the South Jiangsu uplift is referred to as the Dongtai Depression.

The Hongze Sag is situated in the northwest of the Subei Basin, adjoining the Lusu Uplift to the north, the Dengma Fault to the south, the Jianhu Uplift and the Zhangbaling Uplift to the east, and the Huai'an Uplift to the west. Bordered by the Zhenglu Fault Zone, it extends northeastward and represents a Mesozoic and Cenozoic skip-shaped fault depression, thicker in the southern and northern parts. Sedimentation in the sag commenced during the Cretaceous period, reaching its zenith in the Paleogene and Neogene eras, with a cumulative sedimentary thickness of up to 5800 m. Drilling activities in the region have unveiled various formations, including the Upper Cretaceous Pukou Formation, Chishan Formation, Paleogene Taizhou Formation, Funing Formation, Dainan Formation, Sanduo Formation, Neogene Yancheng Formation, and Quaternary Dongtai Formation. The sag is a compound structure characterized by alternating depressions and uplifts. Progressing from southwest to northeast, these are the Jinli sub-sag, the Guanzhen sub-sag, and the Zhaoji sub-sag. Rock salt deposits are found in the fourth section of the Funing Formation within the Zhaoji sub-sag. Conversely, the secondary sag and Guanzhen secondary sag are primarily composed of gypsum-containing mudstone, with no salt rock deposits identified.

Almost complete Upper Cretaceous-Cenozoic strata was uncovered in the Hongze Sag, except for the Oligocene-earliest Pliocene absent caused by uplift erosion^27,28^. The Upper Cretaceous strata consist of the Pukou Formation, Chishan Formation, and Taizhou Formation. The Cenozoic strata include the Paleogene Funing Formation, the Eocene Dainan Formation, and Sanduo Formation, the Neogene Yancheng Formation and the Quaternary Dongtai Formation^29–31^.

The Funing Formation is composed of four stratigraphic members (E_1_f_1_-E_1_f_4_) from bottom to top^32^. The first member (E_1_f_1_) comprises a sequence of fluvial-deltaic deposits, while E_1_f_2_ is characterized by lacustrine deposits, predominantly consisting of dark gray-black mudstones/shales interbedded with marls, limestone, and dolomite. E_1_f_3_ is primarily composed of deltaic deposits^32^. The fourth member (E_1_f_4_) are characterized by thick evaporite rock typified by the halite-rich rock and sulfate salts, with a minor amount of carbonate rock.

### Lithology and stratigraphy of core SB18

The lithology of Core SB18 (Fig. 1B) comprises distinct evaporitic-siliciclastic cycles, representing the lower members of the Funing Formation, which are extensively distributed throughout the Subei Basin. These members primarily consist of evaporites interspersed with carbonate and sulfate layers. The halite sediments in the studied Core SB18 often exhibits orange and gray color. The orange halite is predominantly medium- and fine-grained, whereas the gray halite is mostly fine-grained with a minor presence of anhydrite.

## Materials and experimental procedure

### Materials

**Figure 2:**
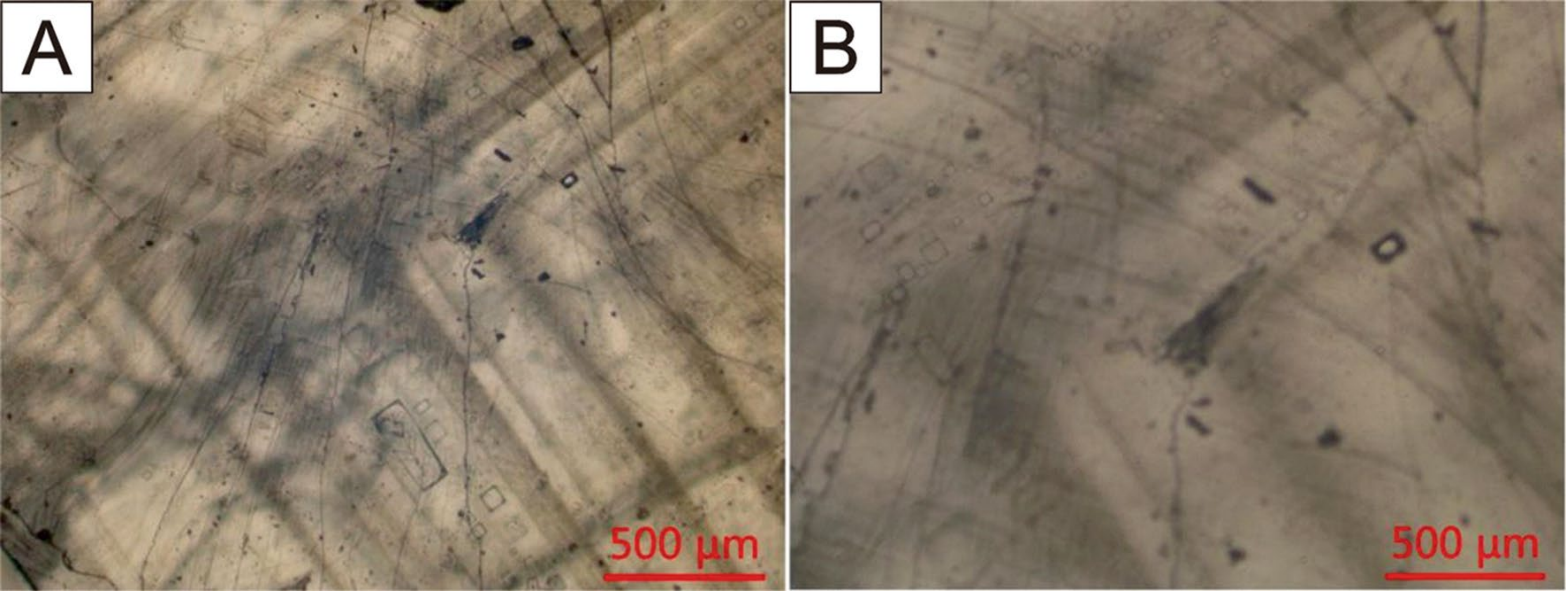
Primary fluid inclusions in the lower member of Funing formation.

The halite sample analyzed in this study was obtained from the depth interval of 1654.87 to 1770.31 m from well SB18, belonging to section E_1_f_4_. The halite samples mainly comprise transparent, gray, or orange halite. Transparent halite crystals exhibit clarity, with few small bands of fluid inclusions. Conversely, gray or white halite samples primarily exhibit fluid inclusion bands. Typically, cumulate crystals coexist with chevron crystals within the same halite beds. Both cumulate and chevron crystals display alternating inclusion-rich and inclusion-poor growth bands (Fig. 2), with cumulate crystals measuring in millimeters and chevron crystals measuring in centimeters, displaying a cloudy appearance. Inclusion-rich bands are characterized by abundant and well-preserved inclusions, appearing cloudy with a white to milk-white color, while bands with few inclusions are often clear and transparent. Generally, primary fluid inclusions range in size from 10 to 60 μm in diameter and are dominated by one-phase aqueous liquid inclusions. Fewer two- or three-phase inclusions, and rare liquid–vapor inclusions, are observed at laboratory temperature. Therefore, only the primary one-phase liquid inclusions within cloudy fluid inclusion bands in chevron and cumulate crystals were selected for homogenization temperature analysis. Transparent halite typically forms diagenetically due to the filling of dissolution cavities by halite cements, displacing the growth of new halite crystals^17^.

### Experimental procedure

Most of the primary inclusions in the studied samples consist of single-phase aqueous liquid inclusions at room temperature, although some contain two-phase inclusions containing gases or solids. Before initiating the ‘cooling nucleation’'^33^ process, we captured photographs of them to differentiate inclusions with gas bubbles at room temperature from those in which vapor bubbles were artificially nucleated after cooling. Previous studies that observed gas bubbles at room temperature before cooling have interpreted these bubbles to be trapped atmospheric air. It is noted that such bubbles can lead to homogenization temperatures that are unrealistically high^14,20,34,35^.

During the preparation process of fluid inclusion sheets, measures were taken to avoid alterations in the original temperature information of the halite inclusions due to cutting, grinding, and polishing. The processing of the inclusion test samples followed the methods outlined by Lowenstein and Bension^17,18^. Initially, a knife was used to cut the halite particles along the cleavage plane to obtain cleavage pieces with a thickness of 1 to 5 mm. These cleavage slices were then examined and photographed under a microscope, with a focus on recording the occurrence and shape of fluid inclusions resulting from primary and early diagenetic recrystallization. Emphasis was placed on photographing single liquid inclusions. Subsequently, the cleavage slices were sealed in plastic zip lock bags and placed in a well-sealed plastic box along with desiccant for protection. The samples were then frozen in a refrigerator for approximately one week (the temperature of the refrigerator was stabilized at -18 °C after multiple measurements), allowing single phases to develop.

All samples were submitted to the MNR Key Laboratory of Metallogeny and Mineral Assessment for homogenization temperature testing. The homogenization temperature was measured after bubbles appeared in the liquid inclusions upon freezing and nucleation. The homogeneous temperature test was conducted using the Linkam THMSG600 hot and cold stage, with a heating rate of 0.5 °C/min, which was then reduced to 0.1 to 0.2 °C/min as the bubbles gradually decreased in size and approached homogeneity.

To ascertain whether the bubbles in the inclusions have genuinely disappeared and reached homogeneity, or if they have merely decreased in size and are challenging to observe under the microscope due to potential inaccuracies, the following method can be employed: Lower the temperature by an additional 10 to 15 °C. If the bubbles persist, their volume will increase once more until they are visible within the field of view. If the bubbles do not reappear even after the fluid inclusion has reached complete homogeneity in temperature and is cooled again by 10 to 15 °C, it indicates that homogeneity has indeed been achieved^34^.

## Results

**Figure 3:**
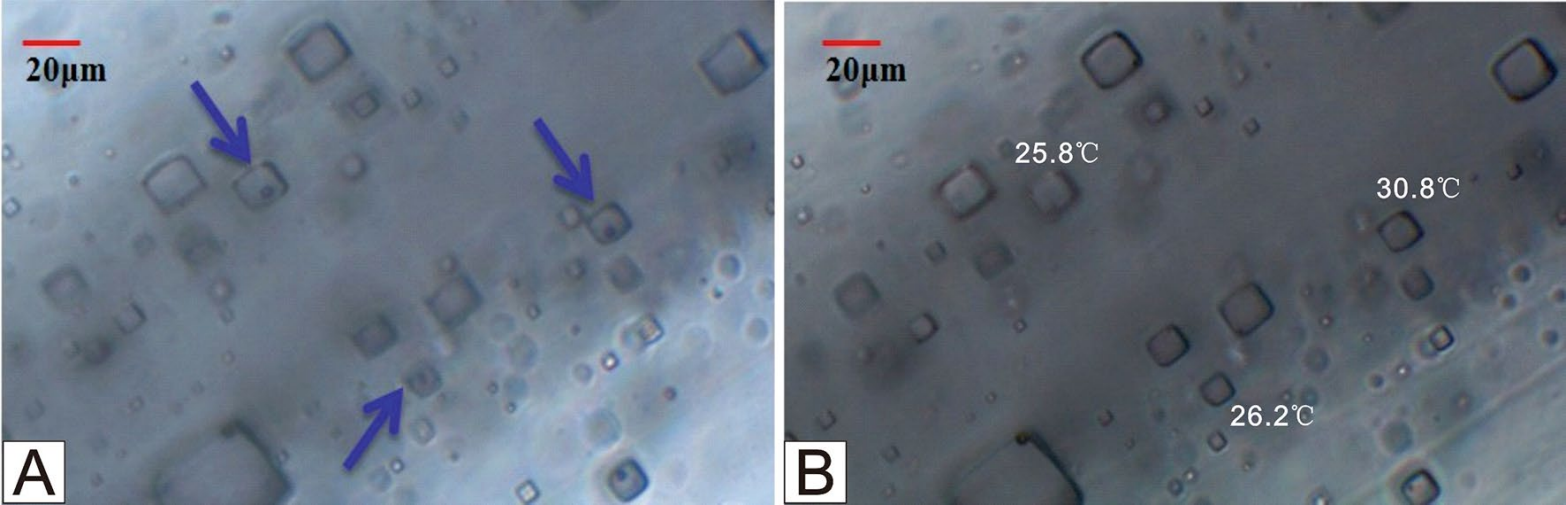
Changes of primary fluid inclusion during the “cooling nucleation process”.

**Figure 4:**
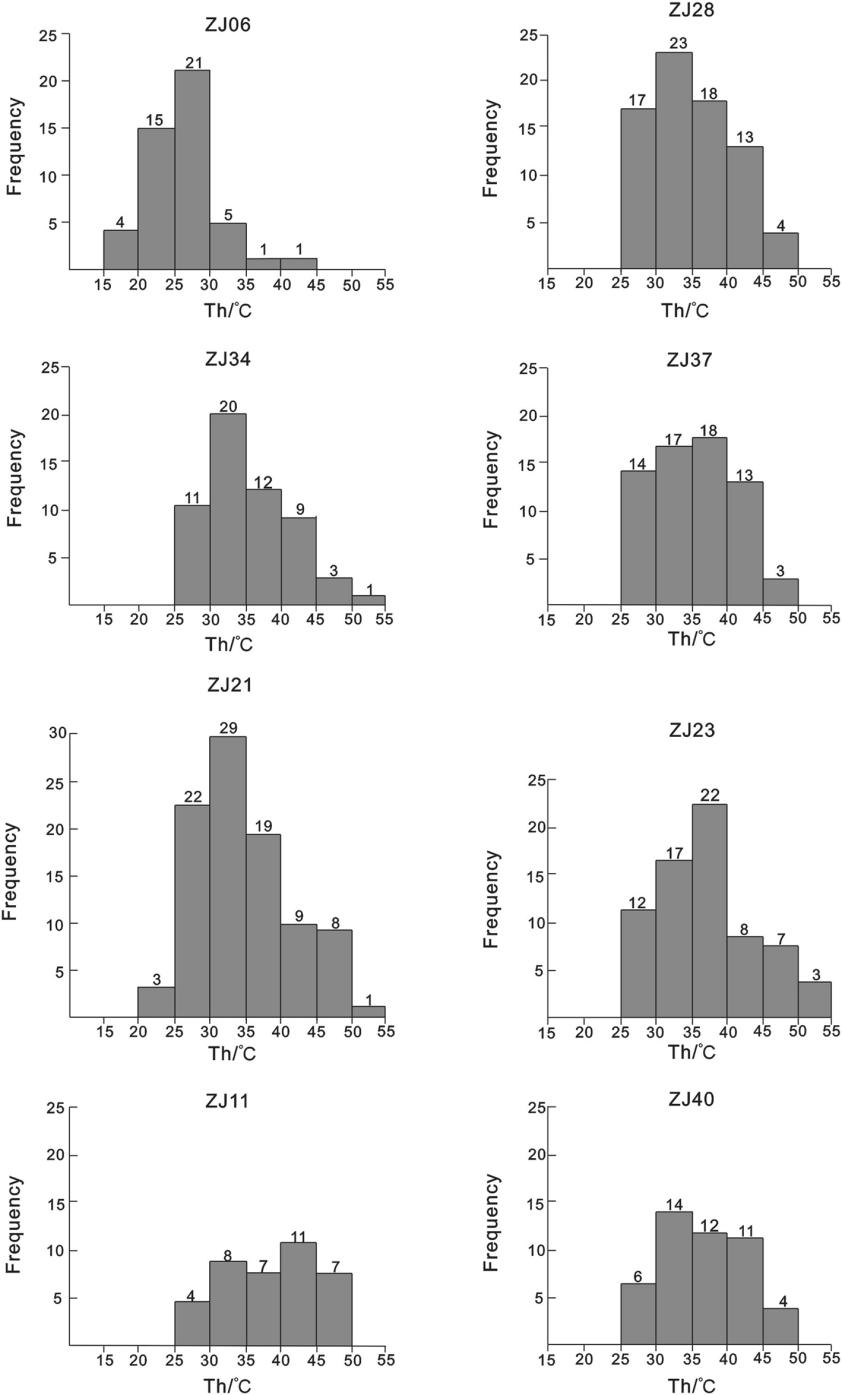
Histogram of homogenization temperature plotted against number of fluid inclusions.

Nucleated vapor bubbles after cooling were observed in less than 15% of primary single-phase liquid fluid inclusions in the halite samples (Fig. 3). The recorded homogenization temperature (Th) data are summarized in Table 1. In total, 488 homogenization temperature data points were obtained from nucleated vapor bubbles in the first round, with a maximum Th of 52.3 °C and a minimum Th of 17.7 °C. Approximately 70% of the Th data fell within the range of 30–50 °C. The Th ranges for the six stratigraphic intervals are as follows: 17.7–41.1 °C (1754.87 m), 21.5–49.2 °C (1756.82 m), 21.2–51.2 °C (1758.61 m), 25.7–51.2 °C (1759.58 m), 26.7–48.7 °C (1760.52 m), 28.1–50.1 °C (1763.57 m), 25.1–45.3 °C (1768.21 m), and 28.4–50.1 °C (1770.31 m), respectively. All samples yielded similar maximum homogenization temperatures or temperature ranges for cumulate crystals and chevron crystals. More than 96% of fluid inclusion assemblages (FIA) had homogenization temperature ranges within 20 °C, and over 90% of FIA had ranges within an even smaller interval of less than 15 °C (more than 90%). Further details of this data are shown in Fig. 4 and listed in Table 1.

**Table 1:** Homogenization temperatures of halite fluid inclusions of the Funing Formation.

Sample	Depth (m)	FIA	Crystal	Th (°C) /size (μm)	Th_max_ (°C)	Th_min_ (°C)	Th_avg_ (°C)
ZJ06	1754.87	FIA 1	Chevron	24/14; 22.8/12; 26.1/15; 27.8/13; 26.9/12;	27.8	22.8	25.52
		FIA 2		20.2/9; 17.7/18; 18.9/19; 20.2/15; 21.8/14; 22.6/12; 24.7/12; 27.9/11;	27.9	17.7	21.75
		FIA 3		22.4/12; 23.4/13; 25.9/15; 26.8/11; 19.6/12;	26.8	19.6	23.62
		FIA 4		20.8/10; 19.8/11; 25.7/15; 27.2/12; 25.6/8; 26.7/16; 28.5/13; 31.5/12;	31.5	19.8	25.73
		FIA 5		22.7/11; 23.9/11; 22.7/12; 26.7/11; 26.7/9; 28.1/16; 29.8/11;	29.8	22.7	25.80
		FIA 6		22.5/16; 21.2/14; 26.1/11; 29.4/19;	29.4	21.2	24.80
		FIA 7	Cumulate	31.2/22; 29.4/21; 32.8/19; 39.4/25; 41.1/22;	41.1	29.4	34.78
		FIA 8	Chevron	27.4/12; 25.8/14; 26.2/11; 30.8/15; 33.4/19	33.4	25.8	28.72
ZJ11	1756.82	FIA 1	Chevron	26.8/12;25.7/8;40.1/23;37.6/19;44.5/26;37.6/18;	44.5	25.7	35.38
		FIA 2		23.2/11;25.8/15; 36.8/21; 37.6/23;	37.6	23.2	30.85
		FIA 3		25.7/15; 32.7/21; 33.4/19; 33.9/18; 34.2/17; 35.9/15	35.9	25.7	32.63
		FIA 4		21.5/12; 22.1/15; 22.8/13; 28.1/19; 28.8/15; 33.6/16; 35.7/17; 38.9/19; 44.2/21; 46.7/20	46.7	21.5	32.24
		FIA 5		25.8/12; 29.4/18; 37.6/19; 44.8/12; 49.2/24	49.2	25.8	37.36
		FIA 6		33.4/16; 34.6/17; 38.9/24; 39.1/26; 39.8/21; 42.1/19	42.1	33.4	37.98
ZJ21	1758.61	FIA 1	chevron	27.8/15;28.6/16;29.7/14;31.2/15;33.6/19;35.4/18;	35.4	27.8	31.05
		FIA 2		29.8/13;30.8/21;31.1/17;31.9/18;32.2/19;33.7/21;37.4/16;39.2/21;41.2/24;44.8/24	44.8	29.8	35.21
		FIA 3		30.1/21;30.4/19;31.2/19;33.4/20;41.2/25;	41.2	30.1	33.26
		FIA 4		21.2/18;22.7/20;28.1/19;30.1/21;31.5/23;33.9/23;38.1/25;44.1/24;45.7/25	45.7	21.2	32.82
		FIA 5		26.8/17;29.1/21;30.8/12;33.9/17;43.1/23;49.2/12;51.2/12	51.2	26.8	37.73
		FIA 6		29.2/18;29.7/14;30.2/18;31.6/21;33.5/21;36.7/23;39.4/23;43.2/13;49.2/15	49.2	29.2	35.86
		FIA 7		28.6/14;29.2/13;32.1/12;33.1/15;35.1/16;39.4/16;42.1/21;49.2/14	49.2	28.6	36.10
		FIA 8		23.1/21;26.1/19;27.2/15;25.3/12;28.1/19;28.6/16;32.1/15;35.7/21;39.2/12	39.2	23.1	29.49
		FIA 9		28.2/12;29.2/13;31.2/15;33.2/21;33.6/12;36.2/15;37.9/15;39.2/16;39.7/18;42.1/15;49.3/13;	49.3	28.2	36.35
		FIA 10		29.1/21; 29.3/15; 31.3/18; 33.2/18; 36.4/16; 38.9/15; 47.1/14; 48.1/12	48.1	29.1	36.68
		FIA 11		29.4/15; 29.9/16; 31.2/15; 32.4/16; 35.7/15; 37.2/21; 39.8/15; 42.3/12; 49.5/16	49.5	29.4	36.38
ZJ23	1759.58	FIA 1	Chevron	25.7/15; 29.7/15; 30.4/14; 31.4/19; 33.5/21; 35.7/12; 36.5/19; 44.8/12; 51.2/13	51.2	25.7	35.43
		FIA 2		26.8/15; 28.1/; 29.4/15; 31.5/15; 36.1/14; 36.7/12; 39.1/15; 45.2/19; 49.6/13	49.6	26.8	35.83
		FIA 3		28.7/15; 29.1/15; 30.1/16; 32.4/15; 33.6/14; 39.1/15; 40.6/14; 44.6/18	44.6	28.7	34.78
		FIA 4		29.1/15; 31.2/15; 33.9/15; 36.4/14; 38.7/14; 39.3/17; 48.1/15; 52.3/13	52.3	29.1	38.63
		FIA 5		30.2/18; 31.8/14; 32.6/15; 36.1/15; 39.7/14; 40.3/15; 44.3/15; 49.1/13	49.1	30.2	38.01
		FIA 6		29.7/14; 30.2/14; 33.1/16; 35.7/14; 37.6/15; 38.3/15; 39.4/15; 42.5/15	42.5	29.7	35.81
		FIA 7		29.7/15; 30.5/14; 33.4/16; 35.7/16; 38.1/14; 39.1/24; 39.4/22; 42.3/21; 46.8/17; 50.9/14	50.9	29.7	38.59
		FIA 8		28.7/15; 29.6/16; 32.4/16; 38.7/15; 37.8/18; 39.6/18; 44.2/16; 47.6/21; 47.8/17	47.8	28.7	38.49
ZJ28	1760.52	FIA 1	Chevron	27.6/15; 28.9/16; 29.1/17; 30.6/14; 33.4/14; 36.4/15; 37.5/18; 42.1/18; 44.3/15	44.3	27.6	34.43
		FIA 2		26.9/15; 29.3/16; 30.2/18; 33.7/16; 35.1/19; 39.4/21; 40.3/19; 49.2/15	49.2	26.9	35.51
		FIA 3		28.4/16; 29.7/18; 31.5/19; 33.7/20; 35.4/15	35.4	28.4	31.74
		FIA 4		26.7/15; 29.4/16; 30.5/19; 35.7/15; 40.1/23; 45.6/24	45.6	26.7	34.67
		FIA 5		29.1/15; 30.1/14; 31.2/15; 33.6/18; 36.7/14; 38.1/19; 42.3/15; 44.6/16	44.6	29.1	35.71
		FIA 6		28.7/17; 29.4/18; 32.4/18; 35.7/16	35.7	28.7	31.55
		FIA 7		30.2/19; 33.8/16; 39.7/18; 40.8/16; 44.2/16; 45.2/17; 48.7/13	48.7	30.2	40.37
		FIA 8		32.1/15; 35.7/18; 38.4/16; 39.1/19; 40.2/19; 42.8/16;	42.8	32.1	38.05
		FIA 9		28.9/14; 29.6/17; 30.5/14; 33.7/14; 35.7/12; 36.7/19; 39.2/21	39.2	28.9	33.47
		FIA 10		29.1/16; 30.7/21; 30.9/19; 32.4/18; 32.4/16; 33.9/17; 35.4/12; 42.5/16	42.5	29.1	33.41
		FIA 11		28.9/15; 29.4/16; 30.1/15; 33.5/15; 36.8/16; 41.2/21; 44.3/18	44.3	28.9	34.89
ZJ34	1763.57	FIA 1	Chevron	28.4/13; 29.4/16; 31.6/18; 33.5/16; 38.7/19; 42.3/17; 46.9/17; 50.1/14	50.1	28.4	37.61
		FIA 2		29.1/14; 30.2/16; 31.5/20; 33.6/17; 33.9/18; 39.4/17; 40.2/18; 43.8/19; 44.9/17;	44.9	29.1	36.29
		FIA 3		30.1/17; 31.8/15; 33.6/16; 37.6/18; 40.1/16; 44.7/18; 45.6/17; 47.9/16	47.9	30.1	38.93
		FIA 4		29.7/17; 28.9/16; 30.2/17; 33.8/17; 35.9/17; 39.7/19; 40.2/19	40.2	29.7	34.06
		FIA 5		28.1/19; 32.5/15; 34.5/15; 36.9/17; 39.5/14	39.5	28.1	34.30
		FIA 6		28.7/18; 29.9/18; 30.2/19; 30.6/17; 32.9/18; 36.1/14; 37.9/19;	37.9	28.7	32.33
		FIA 7		28.9/17; 29.1/17; 30.1/18; 33.8/17; 36.4/16; 39.1/12	39.1	28.9	32.90
		FIA 8		29.1/17; 30.6/18; 31.1/18; 39.4/17; 40.2/17; 42.7/18;	42.7	29.1	35.52
ZJ37	1768.21	FIA 1	Chevron	28.7/18; 30.2/17; 33.1/18; 36.1/17; 39.7/21; 40.2/19	40.2	28.7	34.67
		FIA 2		29.7/21; 32.3/20; 33.8/19; 35.7/17; 38.2/19; 39.2/20; 40.1/21; 44.5/17	44.5	29.7	36.69
		FIA 3		27.9/18; 28.9/19; 31.2/20; 32.3/21; 33.5/20; 36.7/21; 39.1/20; 40.3/20; 42.3/19; 45.2/21	45.2	27.9	35.74
		FIA 4		28.9/16; 30.1/15; 32.1/17; 37.1/15; 39.1/17; 41.3/21; 43.8/19; 45.3/17	45.3	28.9	37.21
		FIA 5		25.1/17; 27.3/20; 29.1/19; 32.1/21; 35.4/19; 37.8/19; 40.6/20; 41.3/20; 42.8/23	42.8	25.1	34.61
		FIA 6		26.4/17; 29.2/15; 30.2/16; 33.2/19; 35.1/18; 36.7/19; 39.2/17;	39.2	26.4	32.86
		FIA 7		27.2/18; 28.1/17; 29.8/17; 30.7/19; 33.2/20; 36.4/19; 39.2/17; 42.6/17; 44.6/15; 47.2/17	47.6	27.2	32.09
		FIA 8		28.3/15; 30.1/17; 30.8/17; 33.2/19; 35.8/19; 39.6/19; 41.3/14; 44.3/20	44.3	28.3	33.91
ZJ40	1770.31	FIA 1	Cumulate	26.9/16; 29.8/17; 32.7/21; 35.6/21; 36.8/17; 39.7/17; 40.9/15; 44.5/19; 50.1/14	50.1	29.6	35.01
		FIA 2		30.2/15; 31.5/17; 32.9/18; 33.9/15; 39.5/18; 41.2/19; 42.3/19	42.3	30.2	35.93
		FIA 3		28.4/19; 30.2/19; 32.1/17; 34.5/18; 36.8/18; 40.1/17; 44.2/17; 46.8/19; 49.2/12	49.2	28.4	35.19
		FIA 4		29.1/14; 32.1/18; 35.7/18; 38.1/19; 39.4/18; 40.2/18; 42.3/19; 45.1/19	45.1	29.1	36.70
		FIA 5		28.7/19; 31.2/19; 33.4/19; 36.1/18; 39.7/19; 40.2/18	40.2	28.7	34.88
		FIA 6		30.8/19; 32.4/19; 33.8/18; 36.1/17; 39.2/17; 40.1/15; 42.1/18	42.1	30.8	36.36

## Discussion

### Rationality and stability of Th data

Due to the deliquescence and solubility of halite, it is prone to damage or recrystallization during burial and preservation, which can affect the reliability and stability of Th data, particularly in the case of ancient halite samples^18^. The Th data do not require pressure correction because halite was deposited in shallow depositional environments, and the temperature trapped by fluid inclusions at low pressure is approximately equal to Th, thereby providing a direct temperature record of halite deposition. Primary fluid inclusions in the growth zone are captured at the same time from the same inclusion. However, in well-developed halite crystals, multiple primary fluid inclusion bands may not form simultaneously, such as those formed in the morning and evening of the same day. Consequently, Th data from different inclusion bands are obtained at different temperatures^18^.

**Figure 5:**
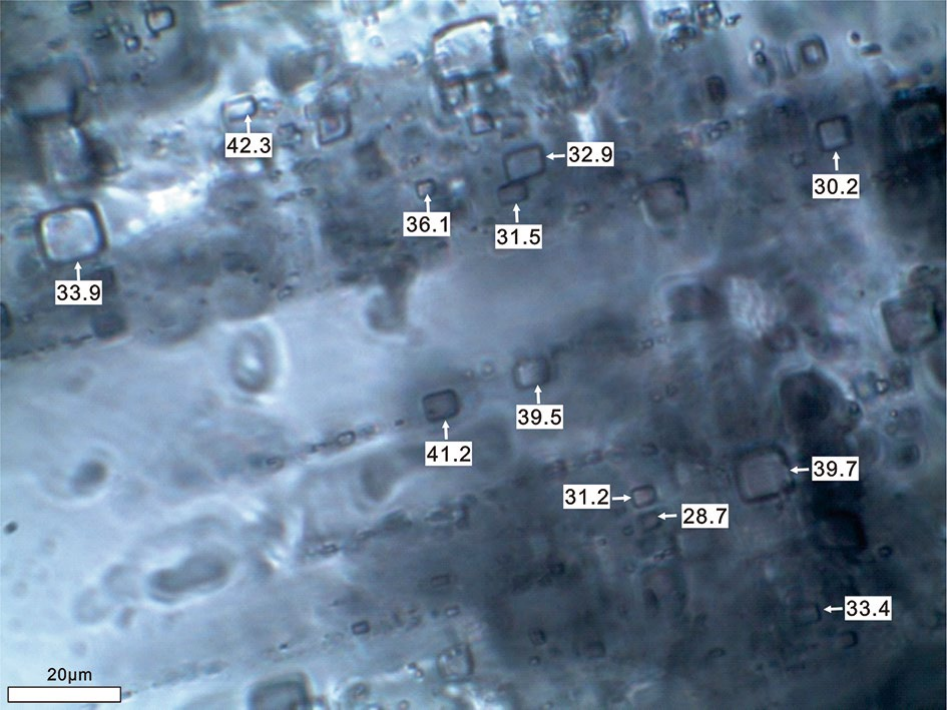
Schematic representation of Th of inclusion bands in sample ZJ40.

We employ two methods to verify whether primary fluid inclusions undergo changes or disruptions in thermal reequilibration, including thermal reequilibration analysis of Th data and examining the relationship between fluid inclusion size and Th. Firstly, the consistency of Th data within a given FIA can serve as an indicator to evaluate thermal reequilibration. As noted by^18^, approximately 90% of Th data within a single FIA fluctuated within a range of less than 15 °C (Fig. 5), indicating that primary fluid inclusions have not experienced changes or disruptions in thermal reequilibration.

**Figure 6:**
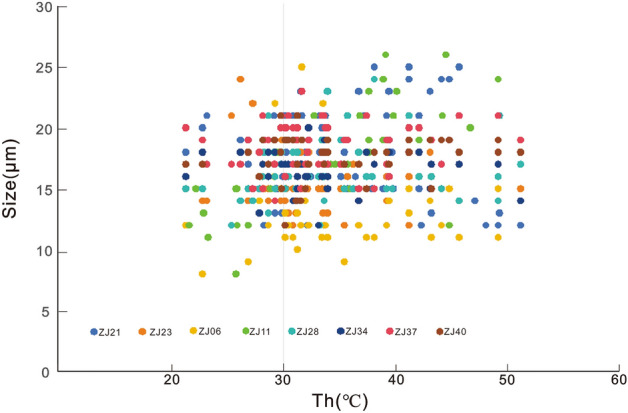
Histogram of homogenization temperatures plotted against size of inclusions.

On the other hand, the relationship between fluid inclusion size and Th can also serve as another criterion for determining possible alteration from thermal reequilibration. Larger inclusions are more extensible than smaller ones, and fluid inclusions that have been subjected to extension tend to exhibit higher Th than their actual capture temperature^36–38^. In other words, if inclusions have experienced extension, larger ones are more likely to yield higher Th compared to smaller ones. However, our results reveal no significant relationship between fluid inclusion size and corresponding Th (Fig. 6). Therefore, the Th data indicate no alteration or damage of our studied halite samples due to thermal reequilibration, further supporting the reliability of the data for paleotemperature interpretations. Consequently, we conclude that the Th data presented here from halite of the Funing Formation likely accurately reflect Late Paleocene water temperatures.

### Record of paleotemperature by halite fluid inclusions

Primary fluid inclusions of the studied halite samples are typically found in both cumulate and chevron crystals. Cumulate crystals often form at the air–water interface or within the upper water column and subsequently settle to the bottom under the influence of gravity^17,25,34^. Chevron crystals, on the other hand, are vertically oriented bottom-growth crystals^38,39^. Our cumulate and chevron halite crystals usually occurred within the same halite beds. The coexistence of cumulate and chevron crystals in the same halite layer generally indicates a shallow water environment that rarely more than a few tens of centimeters and are typically only several centimeters in depth (Lowenstein and Hardie, 1985). Therefore, our obtained homogenization temperatures of fluid inclusions from both cumulate crystals and chevron crystals serve as proxies for brine temperatures or approximate surface air temperatures during halite deposition^17,19,25,39–41^.

Although homogenization temperature data provide insights into brine temperatures, it’s important to acknowledge that they may not capture the complete temperature variation. This limitation arises from the fact that (1) only a small fraction of the millions of fluid inclusions in an individual sample can nucleate vapor bubbles for Th measurements; (2) salt and the fluid inclusions they contain do not form at constant rates over the entire daily and seasonal temperature range. Salt formation typically peaks during daytime, particularly in the afternoon, and during summer months^17,18,34^. In addition, based on petrology and homogenization temperature of halite inclusions from Badenian saline basin, Galamay et al. (2021) proposed that the obtained crystallization temperatures of bottom halite were associated with the depth of crystallization of the sedimentation basin bottom which is closely related to the effect of thermocline. Our studied sample ZJ06 yields a maximum Th of 41.1 °C of cumulate crystals that higher than that of chevron crystals with Th_max_ ranges of 27.8 °C ~ 33.4 °C, implying the bottom temperature was different from the air temperature. It was probably related to bottom crystallization in the evening and night due to sinking of heavier supersaturated surface brine for cooling down^25^. Except for the sample ZJ06, our other samples show a similar Thmax of different halite, with chevron halite of 49.2 °C (sample ZJ11), 51.2 °C (ZJ21), 52.3 °C (ZJ23), 49.2 °C (ZJ28), 50.1 °C (ZJ34), 47.6 °C (ZJ37), respectively, and cumulate halite of 50.1°C (ZJ40). This suggested a prevalent high temperature record of brine or surface air temperature and a slight depth variation when these halites deposited. Moreover, Th data of sample ZJ06 implied that the conditions of sedimentation have changed due to the action of the tectonic factors. The ranges of homogenization temperatures may represent the prevalent temperature conditions during halite deposition^34^.

### Comparison with modern evaporative environments

Robert^34^ reported homogenization temperatures from modern halite precipitated in April to May and August at Badwater Basin, Death Valley, California. These Th fall within two ranges (25–39 °C and 45–49 °C), which closely match the temperature of brine recorded in April to May (20–38 °C) and August (43–50 °C). As discussed above, our results are more likely to reflect seasonal temperature variations and exhibit a similar Th distribution pattern to those reported by Roberts and Spencer.

The absence of Th data from the lowest brine temperature (20 °C) to the lowest Th (25 °C) was interpreted as due to the absence of halite formation at low temperatures and low diurnal evaporative conditions^34^. Thus, the low-temperature ranges in this study are more likely to characterize a low-temperature and strong evaporation environment. Lowenstein^17^ pointed out that only the maximum Th in ‘laboratory-grown halite’^17^ matches the highest temperature of the brine from which the halite was precipitated. Liu^42^ reported both extremely higher and lower Th values than the temperature of brines from both laboratory and natural halite, but the deviations were considered to be due to the sample pretreatment process using a saw for polishing. Generally, extremely low Th values compared to the brine temperature were not often observed in halite samples with proper pretreatments from modern natural settings^34^. However, recent studies show that bottom halite has lower crystallization temperature compared to the air temperature in saline basins with a pronounced thermocline. Although some discrepancies exist in the measured water temperature values from where the halite was precipitated, they are still close to the air temperature^17,18,34,43^. Therefore, the paleotemperature annual fluctuations during the deposition of halite at 1754.87–1770.31 m of E1f4 (56 Ma) in well SB18 in the Subei Basin can be considered to range from 17.7 to 52.3 °C (Table 1). In colder months, the air temperature has varied from 17.7 to 32.1 °C, whereas it has been from 26.8 to 52.3 °C in warmer months. Some of the Th recorded values between 21.75 and 40.37 °C (Fig. 6) reveal a sharp temperature transition from cold to warmer seasons in a year, or possibly few halite precipitation in spring and autumn.

### Local and regional climate conditions during the Late Paleocene

During the Late Paleocene, the characteristics of the plant pollen assemblage *(Ulmipollenites minor; Ulmipollenites; Subtriporopollenites; Quercoidites; Rhoipites; Retitricolpites*) in the Subei Basin indicate that the plants during the deposition period of the Funing Formation were high-temperature tolerant plants, suggesting an arid-hot climate at that time^44–46^. Additionally, analysis of paleomagnetic data during the Funing Formation period of the Subei Basin shows that the average paleolatitude is between 10.5 and 23.9°N, suggesting a location in the low to middle latitude region. Our study of the homogenization temperature of fluid inclusions in halite also indicates that the paleotemperature was high during the Late Paleocene.

## Conclusions

Our research confirmed that paleotemperatures were effectively reconstructed using the method of preliminary cooling of halite samples containing single-phase fluid inclusions, followed by subsequent homogenization of inclusions. In each studied sample, homogenization of inclusions occurred in a narrow temperature range.

We obtained quantitative Late Paleocene paleotemperature records by measuring the Th data of primary fluid inclusions in halite crystals. The average paleotemperature is 34.11 °C, with the highest value being 52.3 °C and the lowest at 17.7 °C. This indicates that the temperature of the ancient salt lake water was relatively high at that time. It can also be inferred from the correspondence between paleowater temperature and paleo air temperature that the climate of the Hongze Sag in the Northern Subei Basin corresponds to the high temperature characteristics during the Paleocene.
